# Supplementary material for: Crykey: Rapid identification of SARS-CoV-2 cryptic mutations in wastewater
Source: Nat Commun. 2024 May 28;15:4545. doi: 10.1038/s41467-024-48334-w (PMC11133379; doi:10.1038/s41467-024-48334-w)
Supplement: Supplementary file 1 — Supplementary Information [file 41467_2024_48334_MOESM1_ESM.pdf]

## Supplementary Figures

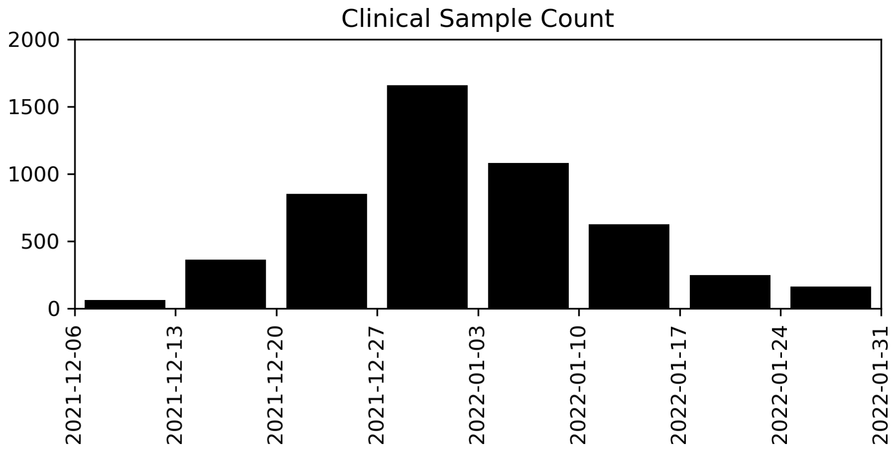


**Supplementary Figure 1. Number of clinical samples used for CR detection during the start of the Omicron season in Houston.** 5,060 sequenced clinical samples collected between 2021-12-06 and 2022-01-31. Each bar represents the number of samples collected during the one week period. The sample count shows a normal distribution pattern where the peak occurs in the week between 2021-12-27 and 2022-01-03.

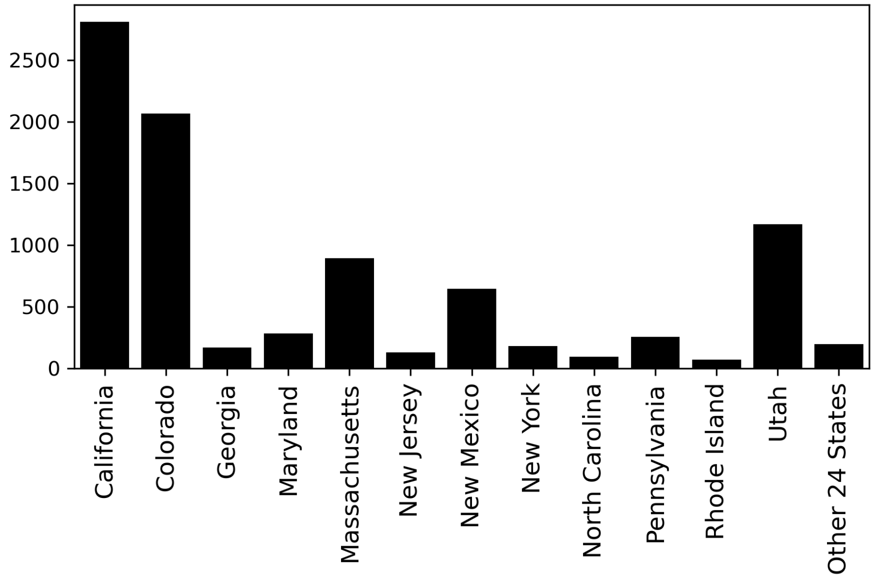


**Supplementary Figure 2. Count of clinical samples used for CR detection during the start of the Omicron season in non-Texas states from the US.** 8,969 sequenced clinical samples were collected between 2021-12-06 and 2022-01-31.

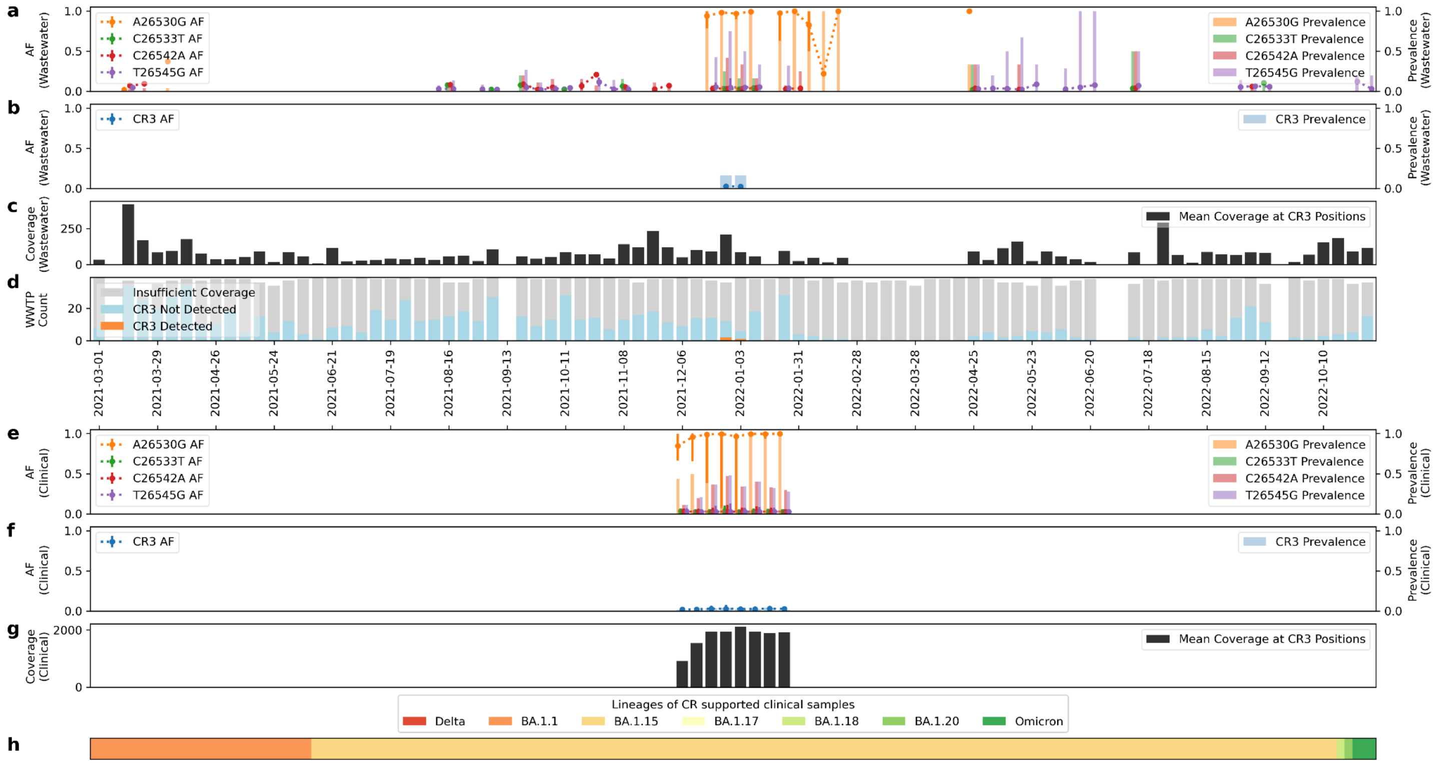


**Supplementary Figure 3. CR3 detected in wastewater and clinical samples from Greater Houston.** Figure a-d are information of CR in wastewater each week, with sample collection date shown in x-axis. a) shows the prevalence rate as bars (left y-axis, same for b, e, f) of each individual mutation within CR in wastewater samples, and the mean AF (right y-axis, same for b, e, f) of each individual mutation presented as dotted line of *n*=90 biologically independent samples for A26530G (shown in orange); *n*=18 for C26533T (shown in green); *n*=34 for C26542A (shown in red) and *n*=71 for T26545G (shown in purple). The error bars (right y-axis, same for b, e, f) are defined as the range of AF, with the top of the error bars showing maximum observed AF, and the bottom of the error bars showing minimum observed AF. b) shows prevalence rate of the CR in wastewater samples, and the mean AF of CR (*n*=3). c) shows the mean coverage at CR3 locations. d) shows the sample qualities and Crykey detections, with samples of insufficient coverage colored in gray, samples of CR absent colored in blue, and samples of CR detected colored in orange. Figure e-h are information of CR in clinical samples of Houston for 8 weeks of sampling period. For figure e-f, the sample collection date is shown in x-axis. e) shows the prevalence rate as bars of each individual mutation within CR in clinical samples, and the mean AF of each individual mutation presented as a dotted line with *n*=4751 for A26530G (shown in orange); *n*=296 for C26533T (shown in green); *n*=1920 for C26542A (shown in red) and *n*=1932 for T26545G (shown in purple). f) shows prevalence rate of CR in clinical samples, and the mean AF of CR (*n*=163). The exact *n* for each individual mutation and CR per week is reported in the Source Data file. g) shows the mean coverage at CR locations. h) shows the distribution of the PANGO lineages of the consensus genomes of the clinical samples with CR. Delta genomes are not found in any of the samples. All Omicron genomes other than BA.1.1, BA.1.15, BA.1.17, BA.1.18, and BA.1.20 are combined and denoted as Omicron. For figure a-c, and e-h, wastewater and clinical samples with insufficient coverage (<10x at CR location) are excluded from the analysis. Source data are available for this figure and are provided in the Source Data file.

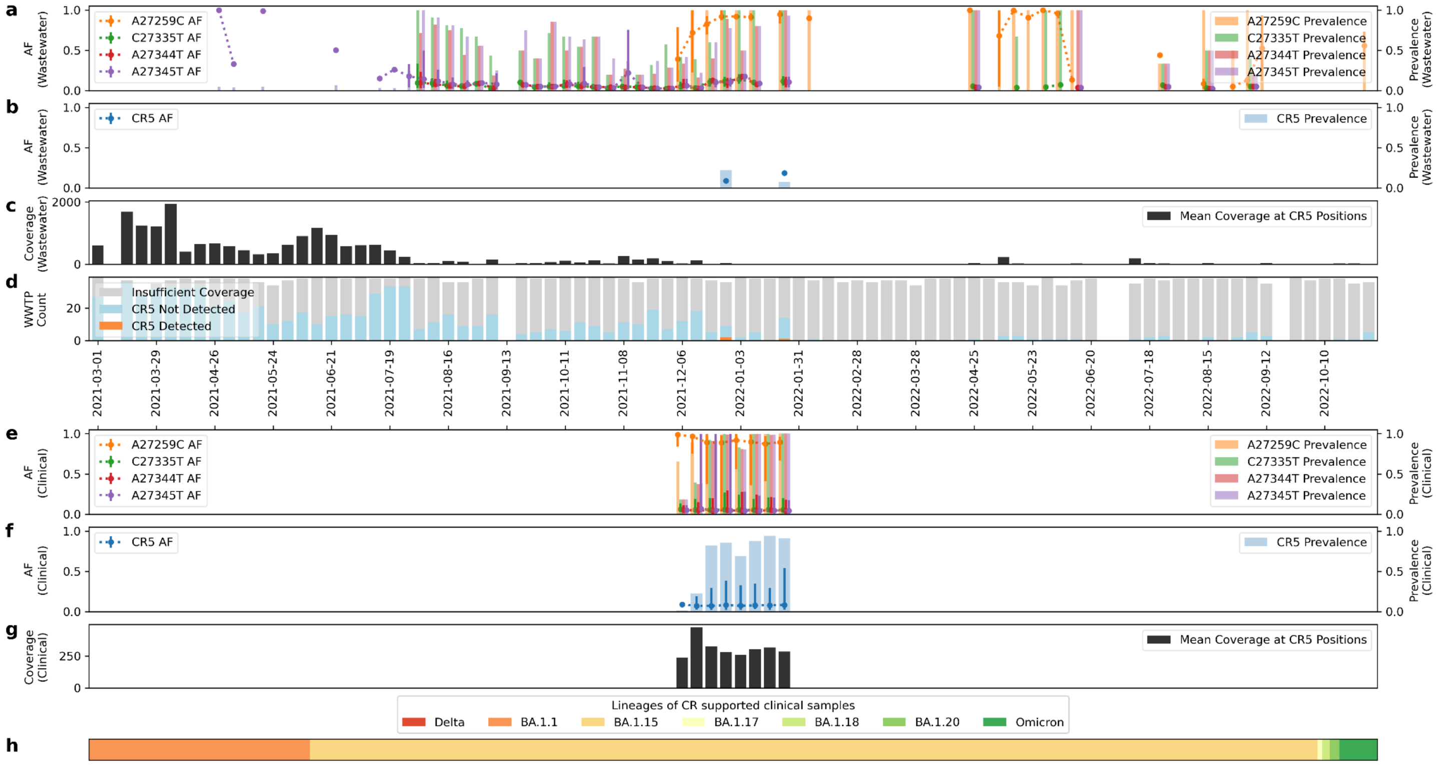


**Supplementary Figure 4. CR5 detected in wastewater and clinical samples from Greater Houston.** Figure a-d are information of CR in wastewater each week, with sample collection date shown in x-axis. a) shows the prevalence rate as bars (left y-axis, same for b, e, f) of each individual mutation within CR in wastewater samples, and the mean AF (right y-axis, same for b, e, f) of each individual mutation presented as dotted line of *n*=81 biologically independent samples for A27259C (shown in orange); *n*=144 for C27335T (shown in green); *n*=116 for A27344T (shown in red) and *n*=146 for A27345T (shown in purple). The error bars (right y-axis, same for b, e, f) are defined as the range of AF, with the top of the error bars showing maximum observed AF, and the bottom of the error bars showing minimum observed AF. b) shows prevalence rate of the CR in wastewater samples, and the mean AF of CR (*n*=3). c) shows the mean coverage at CR5 locations. d) shows the sample qualities and Crykey detections, with samples of insufficient coverage colored in gray, samples of CR absent colored in blue, and samples of CR detected colored in orange. Figure e-h are information of CR in clinical samples of Houston for 8 weeks of sampling period. For figure e-f, the sample collection date is shown in x-axis. e) shows the prevalence rate as bars of each individual mutation within CR in clinical samples, and the mean AF of each individual mutation presented as a dotted line with *n*=4897 for A27259C (shown in orange); *n*=4470 for C27335T (shown in green); *n*=4419 for A27344T (shown in red) and *n*=4416 for A27345T (shown in purple). f) shows prevalence rate of CR in clinical samples, and the mean AF of CR (*n*=3830). The exact *n* for each individual mutation and CR per week is reported in the Source Data file. g) shows the mean coverage at CR locations. h) shows the distribution of the PANGO lineages of the consensus genomes of the clinical samples with CR. Delta genomes are not found in any of the samples. All Omicron genomes other than BA.1.1, BA.1.15, BA.1.17, BA.1.18, and BA.1.20 are combined and denoted as Omicron.

For figure a-c, and e-h, wastewater and clinical samples with insufficient coverage (<10x at CR location) are excluded from the analysis. Source data are available for this figure and are provided in the Source Data file.

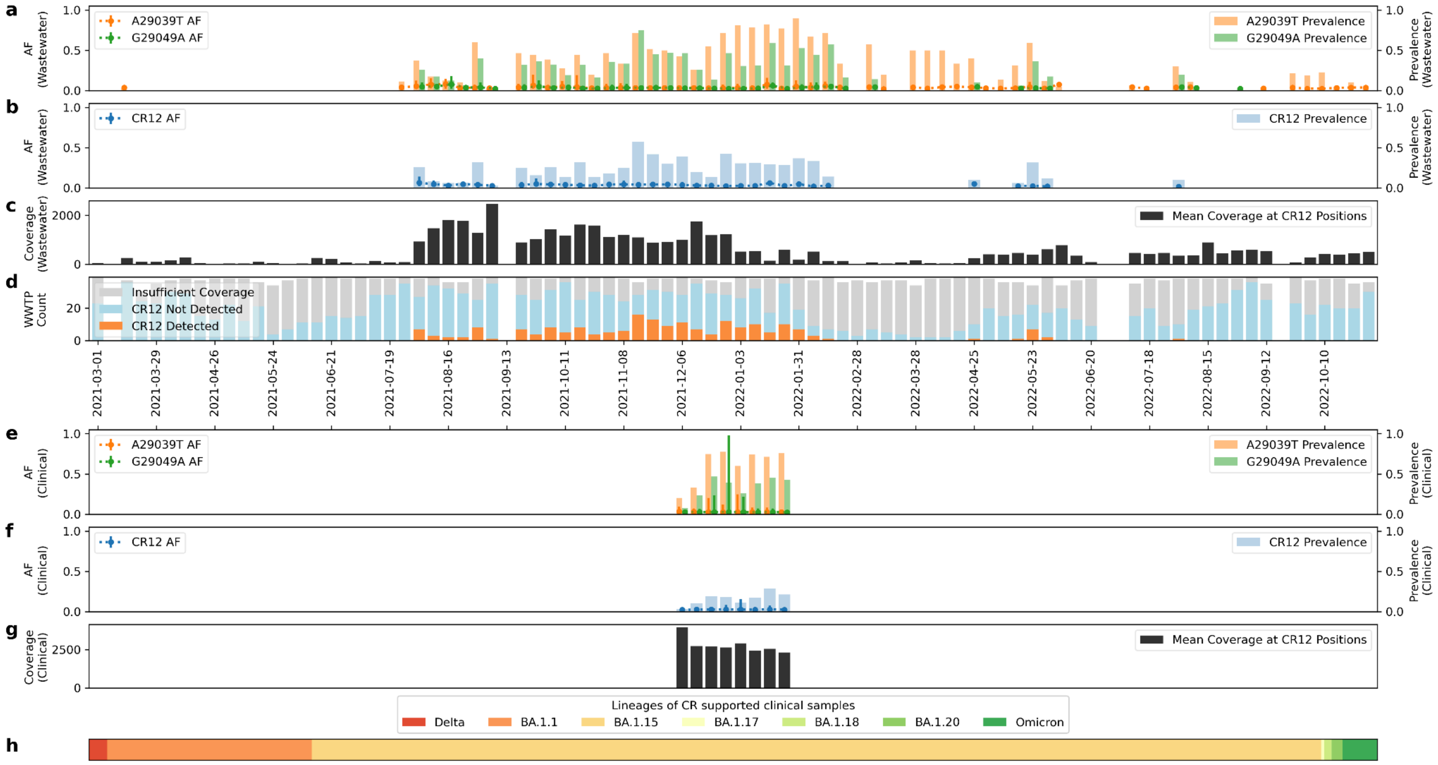


**Supplementary Figure 5. CR12 detected in wastewater and clinical samples from Greater Houston.** Figure a-d are information of CR in wastewater each week, with sample collection date shown in x-axis. a) shows the prevalence rate as bars (left y-axis, same for b, e, f) of each individual mutation within CR in wastewater samples, and the mean AF (right y-axis, same for b, e, f) of each individual mutation presented as dotted line of *n*=416 biologically independent samples for A29039T (shown in orange) and *n*=260 for G29049A (shown in green). The error bars (right y-axis, same for b, e, f) are defined as the range of AF, with the top of the error bars showing maximum observed AF, and the bottom of the error bars showing minimum observed AF. b) shows prevalence rate of the CR in wastewater samples, and the mean AF of CR (*n*=198). c) shows the mean coverage at CR12 locations. d) shows the sample qualities and Crykey detections, with samples of insufficient coverage colored in gray, samples of CR absent colored in blue, and samples of CR detected colored in orange. Figure e-h are information of CR in clinical samples of Houston for 8 weeks of sampling period. For figure e-f, the sample collection date is shown in x-axis. e) shows the prevalence rate as bars of each individual mutation within CR in clinical samples, and the mean AF of each individual mutation presented as a dotted line with *n*=3466 for A29039T (shown in orange) and *n*=1845 for G29049A (shown in green). f) shows prevalence rate of CR in clinical samples, and the mean AF of CR (*n*=845). The exact *n* for each individual mutation and CR per week is reported in the Source Data file. g) shows the mean coverage at CR locations. h) shows the distribution of the PANGO lineages of the consensus genomes of the clinical samples with CR. Delta genomes are not found in any of the samples. All Omicron genomes other than BA.1.1, BA.1.15, BA.1.17, BA.1.18, and BA.1.20 are combined and denoted as Omicron. For figure a-c, and e-h, wastewater and clinical samples with insufficient coverage (<10x at CR location) are excluded from the analysis. Source data are available for this figure and are provided in the Source Data file.

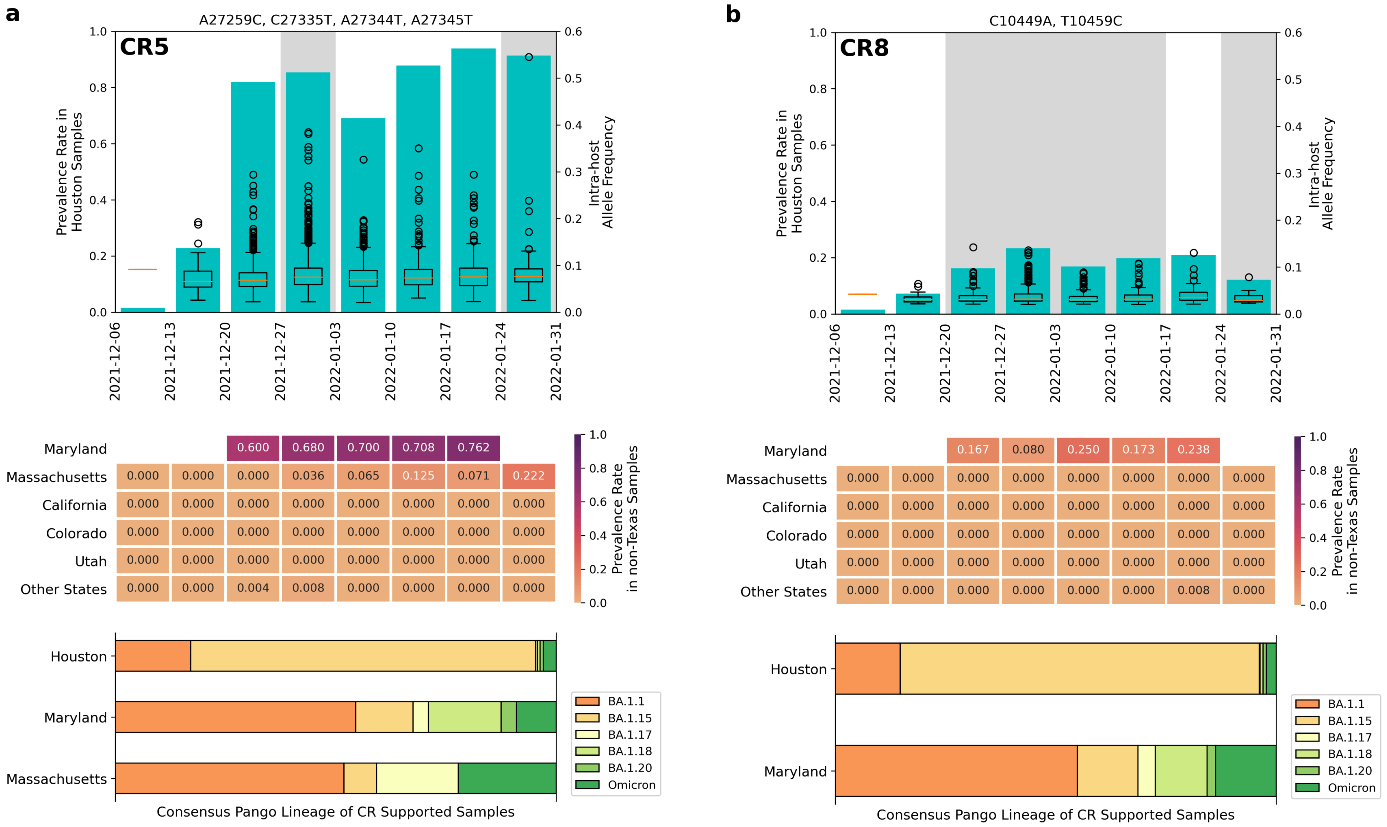


**Supplementary Figure 6. CRs detected in clinical samples from Houston and other US cities.** The figure shows detailed information of a) CR A27259C-C27335T-A27344T-A27345T (CR5) and b) CR C10449A-T10459C (CR8), which are two CRs detected in Houston wastewater that were supported by clinical samples from Houston as well as from other US cities. For each of the CRs, the top figure shows the prevalence rate in Houston clinical samples in bars and the intra-host allele frequency of the CR in supported clinical samples in the box plot (with *n*=1,82,695,1378,744,549,232,149 for CR5 and *n*=1,26,138,386,182,124,52,20 for CR8). The box plot includes both median lines (solid), and the box bounds the interquartile range (IQR). The Tukey-style whiskers extend from the box by at most 1.5 × IQR. The outliers are shown. The center heatmap shows the prevalence rate with colors in other US regions, where white cells indicate no data. The bottom figure shows the distribution of the PANGO lineages of the consensus genomes of the CR in supported clinical samples in Houston and other US regions using different colors. All PANGO lineages of Omicron other than BA.1.1, BA.1.15, BA.1.17, BA.1.18 and BA.1.20 are combined and denoted as Omicron.

### Supplementary Tables

| | Texas | Non-Texas | Combined |
|---|---|---|---|
| **Total Sample Count** | 2,458 | 4,655 | 7,113 |
| **Supported Sample Count** | 28 | 17 | 45 |
| **Prevalence Rate** | 0.0114 | 0.0037 | 0.0063 |
| **Allele Freq Max** | 0.0417 | 0.0185 | 0.0417 |
| **Allele Freq Mean** | 0.0064 | 0.0047 | 0.0057 |
| **Collection Date Start** | 2021-11-06 | 2022-01-24 | 2021-11-06 |
| **Collection Date End** | 2022-03-21 | 2022-02-13 | 2022-03-21 |

**Supplementary Table 1. Pacbio Clinical Samples Supporting CR12 (A29039T-G29049A).** A total of 7113 Pacbio HiFi clinical samples were selected from NCBI SRA database and the sequencing reads were aligned to the reference genome of SARS-CoV-2. The table shows the distribution of samples collected in Texas and other US states containing reads supporting CR A29039T-G29049A, as well as the prevalence rate, max and mean allele frequency of the CR, and the range of sample collection dates.

| | Nucleotide Mutation | Prevalence in Wastewater Samples | Prevalence in Clinical Samples | Consensus Mutation | Translation | Sequences with the AA change | Reported Countries |
|---|---|---|---|---|---|---|---|
| CR1 | A26530G(*) | High | High | Yes | M:D3G | 2.29M | 201 |
| | C26577G(*) | High | High | Yes | M:Q19E | 7.65M | 206 |
| | G26634A | Low | Low | No | M:A38T | 15 | 8 |
| CR2 | C6402T | High | Low | Yes | ORF1a:P2046L | 3.96M | 200 |
| | G6456A | Medium | Medium | No | ORF1a:C2064Y | 436 | 29 |
| CR3 | A26530G(*) | High | High | Yes | M:D3G | 2.29M | 201 |
| | C26533T | Low | Low | No | M:S4F | 6004 | 104 |
| | C26542A | Medium | Medium | No | M:T7N | 212 | 31 |
| | T26545G(*) | Medium | Medium | No | M:I8S | 67 | 22 |
| CR4 | A26530G(*) | High | High | Yes | M:D3G | 2.29M | 201 |
| | T26545G(*) | Medium | Medium | No | M:I8S | 67 | 22 |
| CR5 | A27259C | High | High | Yes | ORF6:R20R | 5.74M | 206 |
| | C27335T | Medium | High | No | ORF6:T45I | 1632 | 67 |

| | | | | | | | |
|---|---|---|---|---|---|---|---|
| | A27344T(*) | Medium | High | No | ORF6:K48I | 154 | 26 |
| | A27345T(*) | Medium | High | No | | | |
| CR6 | T29029C | Medium | Low | No | N:A252A | 3045 | 75 |
| | A29039T | Medium | High | No | N:K256* | 118 | 12 |
| CR7 | A26530G(*) | High | High | Yes | M:D3G | 2.29M | 201 |
| | C26577G(*) | High | High | Yes | M:Q19E | 7.65M | 206 |
| | C26625A | Medium | Low | No | M:L35I | 25 | 7 |
| CR8 | C10449A | High | High | Yes | ORF1a:P3395H | 7.96M | 207 |
| | T10459C | Medium | Low | No | ORF1a:T3398T | 493 | 46 |
| CR9 | T15682A | Medium | Low | No | ORF1b:Y739N | 20 | 8 |
| | T15685A | Medium | Low | No | ORF1b:L740M | 1145 | 31 |
| CR10 | A24966T | Medium | Low | No | S:N1135I | 42 | 7 |
| | C25000T | High | High | Yes | S:D1146D | 7.90M | 207 |
| CR11 | A27344T(*) | Medium | High | No | ORF6:K48I | 154 | 26 |
| | A27345T(*) | Medium | High | No | | | |
| | A27354G | Medium | High | No | ORF6:Q51Q | 3288 | 72 |
| CR12 | A29039T(*) | Low | Medium | No | N:K256* | 118 | 12 |
| | G29049A | Low | Medium | No | N:R259Q | 153 | 34 |

**Supplementary Table 2. Individual mutations of CR1-CR12.** This table contains the information of the prevalence rate of each individual mutation of CR1-CR12 in Houston wastewater and clinical samples, as well as the corresponding amino acid changes of the mutations. We further queried each amino acid change with outbreak.info, and the table shows the number of sequences containing each of the amino acid change around the world (as of 2023-11-02), as well as the number of countries that the amino acid change has been reported. Nucleotide mutation that have been repeatedly found in multiple CRs are marked with (*).

**Supplementary Table 3. GISAID EPI_SET_240326gr**

## Data Availability

GISAID Identifier: EPI_SET_240326gr
doi: 10.55876/gis8.240326gr

All genome sequences and associated metadata in this dataset are published in GISAID's EpiCoV database. To view the contributors of each individual sequence with details such as accession number, Virus name, Collection date, Originating Lab and Submitting Lab and the list of Authors, visit 10.55876/gis8.240326gr

## Data Snapshot

- EPI_SET_240326gr is composed of 13,875,227 individual genome sequences.
- The collection dates range from 2019-12-24 to 2023-01-05;
- Data were collected in 217 countries and territories;
- All sequences in this dataset are compared relative to hCoV-19/Wuhan/WIV04/2019 (WIV04), the official reference sequence employed by GISAID (EPI_ISL_402124). Learn more at https://gisaid.org/WIV04.

## Supplementary Information

### ST1. CRs may contain signal of recombination events

Most of the CRs we found in Houston wastewater contain signature mutations that are specific to only one PANGO lineage, with a few exceptions. A combination of A29301G (N:D343G) and G29402T (N: D377Y) was detected in 22 of the wastewater treatment plants between 2021-12-23 and 2022-01-06. Based on the definition of signature mutations, A29301G (N:D343G) is specific to Omicron BA.1.15, with more than 97% of the reported BA.1.15 (and its descendants BA.1.15.1, BA.1.15.2, and BA.1.15.3) genomes in GISAID EpiCoV containing such mutation. On the other hand, multiple Delta strains in the database, including AY.29.1, AY.9.21, etc, present the G29402T (N: D377Y) mutation. This rare observation may be hinting at a potential recombination event between early Omicron strains and Delta strains of SARS-CoV-2. However, confirming the emergence of recombinant strains requires an examination on mutations across much larger regions of the SARS-CoV-2 genomes, which can not be acquired via wastewater surveillance.
