# Supplementary material for: Crykey: Rapid identification of SARS-CoV-2 cryptic mutations in wastewater
Source: Nat Commun. 2024 May 28;15:4545. doi: 10.1038/s41467-024-48334-w (PMC11133379; doi:10.1038/s41467-024-48334-w)
Supplement: Supplementary file 3 — Reporting Summary [file 41467_2024_48334_MOESM3_ESM.pdf]

nature portfolio

Corresponding author(s): Todd J. Treangen

Last updated by author(s): Apr 5, 2024

Reporting Summary

Nature Portfolio wishes to improve the reproducibility of the work that we publish. This form provides structure for consistency and transparency in reporting. For further information on Nature Portfolio policies, see our Editorial Policies and the Editorial Policy Checklist.

Statistics

For all statistical analyses, confirm that the following items are present in the figure legend, table legend, main text, or Methods section.

n/a	Confirmed	
☐	☒	The exact sample size (*n*) for each experimental group/condition, given as a discrete number and unit of measurement
☒	☐	A statement on whether measurements were taken from distinct samples or whether the same sample was measured repeatedly
☐	☒	The statistical test(s) used AND whether they are one- or two-sided<br>*Only common tests should be described solely by name; describe more complex techniques in the Methods section.*
☒	☐	A description of all covariates tested
☒	☐	A description of any assumptions or corrections, such as tests of normality and adjustment for multiple comparisons
☐	☒	A full description of the statistical parameters including central tendency (e.g. means) or other basic estimates (e.g. regression coefficient) AND variation (e.g. standard deviation) or associated estimates of uncertainty (e.g. confidence intervals)
☐	☒	For null hypothesis testing, the test statistic (e.g. *F*, *t*, *r*) with confidence intervals, effect sizes, degrees of freedom and *P* value noted<br>*Give P values as exact values whenever suitable.*
☒	☐	For Bayesian analysis, information on the choice of priors and Markov chain Monte Carlo settings
☒	☐	For hierarchical and complex designs, identification of the appropriate level for tests and full reporting of outcomes
☒	☐	Estimates of effect sizes (e.g. Cohen's *d*, Pearson's *r*), indicating how they were calculated

Our web collection on statistics for biologists contains articles on many of the points above.

Software and code

Policy information about availability of computer code

Data collection	Data is generated using customized code. The code is available on github: https://github.com/treangenlab/crykey<br>The version of the code we used in this study is also archived at: 10.5281/zenodo.10909155
Data analysis	Data is analyzed using customized code. The code is available on github: https://github.com/treangenlab/crykey_analysis_scripts<br>The version of the code we used in this study is also archived at: 10.5281/zenodo.10909142

For manuscripts utilizing custom algorithms or software that are central to the research but not yet described in published literature, software must be made available to editors and reviewers. We strongly encourage code deposition in a community repository (e.g. GitHub). See the Nature Portfolio guidelines for submitting code & software for further information.

Data

Policy information about availability of data

All manuscripts must include a data availability statement. This statement should provide the following information, where applicable:

- Accession codes, unique identifiers, or web links for publicly available datasets
- A description of any restrictions on data availability
- For clinical datasets or third party data, please ensure that the statement adheres to our policy

Source data is provided with this paper and has been deposited in the OSF database with DOI: 10.5281/zenodo.10934124. All sequencing data supporting the findings of this study is publicly available. Houston wastewater datasets are available for download via NCBI BioProject PRJNA796340. Houston short read clinical

datasets are available for download via NCBI BioProject PRJNA764181. Non-Texas short read clinical datasets are available for download via NCBI BioProject PRJNA686984. The PacBio SARS-CoV-2 clinical datasets are available for download via NCBI BioProject PRJNA718231. There are no restrictions on data availability.

Research involving human participants, their data, or biological material

Policy information about studies with human participants or human data. See also policy information about sex, gender (identity/presentation), and sexual orientation and race, ethnicity and racism.

Reporting on sex and gender	N/A
Reporting on race, ethnicity, or other socially relevant groupings	N/A
Population characteristics	N/A
Recruitment	N/A
Ethics oversight	N/A

Note that full information on the approval of the study protocol must also be provided in the manuscript.

Field-specific reporting

Please select the one below that is the best fit for your research. If you are not sure, read the appropriate sections before making your selection.

☒ Life sciences ☐ Behavioural & social sciences ☐ Ecological, evolutionary & environmental sciences

For a reference copy of the document with all sections, see nature.com/documents/nr-reporting-summary-flat.pdf

Life sciences study design

All studies must disclose on these points even when the disclosure is negative.

Sample size	3,175 short-read Illumina SRA runs of SARS-CoV-2 wastewater samples collected from Houston WWTP between February 2021 and November 2022,<br>5,060 short-read Illumina SRA runs of SARS-CoV-2 clinical samples collected within the Greater Houston between 2021-12-06 and 2022-01-31,<br>8,969 short-read Illumina SRA runs of SARS-CoV-2 clinical samples collected outside of Texas between 2021-12-06 and 2022-01-31,<br>7,113 long-read PacBio SRA runs of SARS-CoV-2 clinical samples collection dates between 2021-11-06 and 2022-03-21.
Data exclusions	There are no data being excluded in this study.
Replication	All the computational experiments are replicated at least twice.
Randomization	The samples are allocated into groups based on the sampling locations and sample collection dates (binning into weekly group). Randomization is not applicable for this study since the software is designed to search potential cryptic lineages based on presence of signal across temporal and spatial samples.
Blinding	Researchers are not blinded during data collection and analysis, because the identification of the group (WWTP/sample collection week) is used as an input for the software to identify potential cryptic lineages.

Reporting for specific materials, systems and methods

We require information from authors about some types of materials, experimental systems and methods used in many studies. Here, indicate whether each material, system or method listed is relevant to your study. If you are not sure if a list item applies to your research, read the appropriate section before selecting a response.

Materials & experimental systems

n/a	Involved in the study
☒	☐ Antibodies
☒	☐ Eukaryotic cell lines
☒	☐ Palaeontology and archaeology
☒	☐ Animals and other organisms
☒	☐ Clinical data
☒	☐ Dual use research of concern
☒	☐ Plants

Methods

n/a	Involved in the study
☒	☐ ChIP-seq
☒	☐ Flow cytometry
☒	☐ MRI-based neuroimaging

Plants

Seed stocks	N/A
Novel plant genotypes	N/A
Authentication	N/A
